# Tree Species Composition and Harvest Intensity Affect Herbivore Density and Leaf Damage on Beech, *Fagus sylvatica*, in Different Landscape Contexts

**DOI:** 10.1371/journal.pone.0126140

**Published:** 2015-05-04

**Authors:** Jule Mangels, Nico Blüthgen, Kevin Frank, Fabrice Grassein, Andrea Hilpert, Karsten Mody

**Affiliations:** 1 Ecological Networks, Department of Biology, Technische Universität Darmstadt, Darmstadt, Germany; 2 Institute of Plant Sciences, University of Bern, Bern, Switzerland; Helmholtz Centre for Environmental Research (UFZ), GERMANY

## Abstract

Most forests are exposed to anthropogenic management activities that affect tree species composition and natural ecosystem processes. Changes in ecosystem processes such as herbivory depend on management intensity, and on regional environmental conditions and species pools. Whereas influences of specific forest management measures have already been addressed for different herbivore taxa on a local scale, studies considering effects of different aspects of forest management across different regions are rare. We assessed the influence of tree species composition and intensity of harvesting activities on arthropod herbivores and herbivore-related damage to beech trees, *Fagus sylvatica*, in 48 forest plots in three regions of Germany. We found that herbivore abundance and damage to beech trees differed between regions and that – despite the regional differences - density of tree-associated arthropod taxa and herbivore damage were consistently affected by tree species composition and harvest intensity. Specifically, overall herbivore damage to beech trees increased with increasing dominance of beech trees – suggesting the action of associational resistance processes – and decreased with harvest intensity. The density of leaf chewers and mines was positively related to leaf damage, and several arthropod groups responded to beech dominance and harvest intensity. The distribution of damage patterns was consistent with a vertical shift of herbivores to higher crown layers during the season and with higher beech dominance. By linking quantitative data on arthropod herbivore abundance and herbivory with tree species composition and harvesting activity in a wide variety of beech forests, our study helps to better understand the influence of forest management on interactions between a naturally dominant deciduous forest tree and arthropod herbivores.

## Introduction

Most forests have been considerably modified by human activities [1–3]. Anthropogenic influences affect forest ecosystems indirectly via activities including hunting, livestock grazing, environmental pollution, human-assisted biological invasions and climate change, but also directly via forest management activities [4–6]. Whereas it is evident that forest management such as logging and reforestation with mono- and polycultures of native or non-native tree species can affect forest biodiversity (sensu [7, 8]) and related ecosystem processes [9–11], the extent of these effects is more difficult to predict and supposedly contingent on the intensity of forest management and on the processes studied [12–15].

Herbivory, the consumption of plant material by animals, is a fundamental ecosystem process that affects nutrient cycles and plant abundance, and as a consequence resources and habitats for other organisms [16–18]. In forests, arthropod herbivores are abundant and diverse [19–21], and both abundance and diversity of herbivores are assumed to contribute to the extent of herbivory in forests [22–24], although the relationship between herbivore abundance, herbivore diversity and herbivory may also be ambiguous [25–27].

Herbivore diversity and abundance on host plants is modified by an array of different factors such as abiotic conditions [28, 29], the quality of host plants [30–32], and the exposure to competitors and natural enemies [33–35]. In forests, these factors may show strong vertical stratification within individual trees [36], and they may also be strongly influenced by tree species composition [37, 38]. Following the vertical stratification of resources and conditions within tree crowns, many arthropod herbivores show a stratified spatial distribution in the canopy [36, 39, 40]. Although a distinct stratification of arthropods has been described for both tropical and temperate forests, stratification seems to be no static characteristic of canopy arthropod assemblages, but it may vary between seasons and with tree species identity, tree age and tree neighborhood [36, 41, 42].

Tree neighborhood reflects age structure and species composition of neighbor trees and can thus be viewed as one aspect of the more general influence of forest management and tree species composition (tree association) on the abundance and diversity of herbivores on forest trees. Although plants in pure stands may often suffer from stronger herbivory than those in mixed stands, both positive and negative effects of plant association on herbivore damage have been reported [43–45]. Plant associations can increase or decrease herbivore density or herbivore damage and are referred to as "associational susceptibility" (AS) and "associational resistance" (AR) respectively [37]. In forest systems, both AS and AR have been documented as a consequence of tree association, which is most commonly measured as tree diversity or dominance of a focal tree species [38, 46]. For example, insect herbivory on oaks and alders was lower in monocultures, whereas herbivory on birch was higher in pure birch stands [22]. To explain such contrasting effects of plant association on plant damage, different hypotheses have been established. These hypotheses consider both bottom-up and top down processes (see [47, 48] for review of associational resistance hypotheses), and the influence of variation in species characteristics [45, 49].

Tree association in forests is strongly determined by forest management, which influences tree species composition and quality [50, 51], and thus directly and indirectly affects the abundance of herbivores [52, 53]. Besides determining tree association, forest management involves harvesting practices, which may, for example, change forest microclimate, host plant quality, host plant quantity, and enemy pressure [4, 54]. As a consequence of these changes in the abiotic and biotic environment, harvesting can have intensity-dependent effects on herbivores and on related damage to trees [55–57]. These effects may either decrease or increase herbivore populations and herbivore diversity depending on species characteristics such as dependence on specific hosts and successional stages of forest [58, 59]. In addition to the potentially strong influence of forest management on herbivore populations and herbivore diversity, all the effects should be regarded in the context of larger-scale environmental variation (including climate, edaphic conditions, landscape heterogeneity) and regional species pools. These factors can have a strong impact on herbivore abundance and damage, and they may dilute, overlay or interact with the effects of forest management [55, 60, 61].

Considering the varying and interacting effects of tree association, harvest intensity and regional environmental conditions on herbivores and their damaging activity, a simultaneous assessment of these factors appears necessary to achieve generalizable information on the major determinants of herbivory in forest ecosystems. In our study, we investigated herbivore density and damage to European beech trees (*Fagus sylvatica* L.) growing in forests with different management histories in three regions of Germany, to clarify the role of forest management on herbivore abundance and on related damage to trees across a variety of silvicultural and abiotic conditions. We focused on beech forests since European beech is the dominating tree species of the potential natural vegetation in Central Europe [62, 63]. Despite their dominant role in European forests, beech trees appear to suffer relatively low damage and to host a less speciose and abundant arthropod assemblage than other common deciduous trees such as oak and maple [64–66]. As increasing abundance and species-area relationships are generally related to increasing species richness [64, 67], the relatively low infestation and species richness of arthropod herbivores on beech emphasizes the need for investigations on determinants of herbivore-beech interactions.

Specifically, we investigated whether (1) herbivore abundance and damage vary between regions differing in various environmental conditions for the same forest type (beech forest) and whether (2) forest management, estimated by beech dominance and harvest intensity, affects herbivore abundance and damage under consideration of expected regional effects. In addition to regional patterns, we (3) also considered herbivore distribution and damage by specific arthropod taxa within tree crowns [14].

## Material and Methods

### Ethics statements

Permits for field work were issued by the responsible state environmental offices of Brandenburg, Thüringen and Baden-Württemberg (according to § 72 BbgNatSchG). The study sites comprise state forests and protected areas such as the National Park Hainich and some nature reserves within the biosphere reserves Schwäbische Alb and Schorfheide-Chorin, as well as in the forest of Keula, Hainich-Dün. No species that are protected by European or national laws were sampled during this study.

### Study area

The study was conducted in the framework of the German Biodiversity Exploratories Project (http://www.biodiversity-exploratories.de). The Biodiversity Exploratories Project addresses effects of land use on biodiversity and biodiversity-related ecosystem processes [7].

Leaf damage by herbivores was assessed in November 2012 (year 1) from fallen leaves and in May and July 2013 (year 2) from live leaves. The assessments in year 2 were also used to quantify herbivore load on study trees. Assessments were carried out in forest plots in the three Biodiversity Exploratories (from north to south) “Schorfheide-Chorin” (SCH; a glacial formed landscape in North-East Germany, 3–140 m a.s.l., 13°23’27”–14°08’53” E / 52°47’25”–53°13’26” N), “Hainich” (HAI; a hilly region in Central Germany, 285–550 m a.s.l., 10°10’24”–10°46’45” E / 50°56’14”–51°22’43” N), and “Schwäbische Alb” (ALB; a low-mountain range in South-West Germany, 460–860 m a.s.l., 09°10’49”–09°35’54” E / 48°20’28”–48°32’02” N). SCH is characterized by the lowest annual precipitation (520–580 mm), with a mean annual temperature of 6–7°C. It is followed by HAI (630–800 mm, 6.5–8°C) and ALB (800–930 mm, 8–8.5°C). More details on the Biodiversity Exploratories can be found in Fischer *et al*. (2010) [7].

### Sample processing

In year 1, fallen leaves were collected on each corner of 15 plots in HAI and in ALB Exploratories. These plots included three different forest types (n = 5 plots per type and region): uncultivated, young and old beech forest. Leaf damage was then calculated as the percentage of leaf damage for 50 leaves randomly chosen per corner that were scanned and afterwards subjected to a pixel analysis using the software Image J [68]. Percent leaf damage was calculated by estimating the number of pixels of the leaf area missing due to herbivory (attributable to chewing herbivores, not mines and galls) and by relating this number to the number of pixels of the whole leaf including intact and damaged leaf parts [69]. In year 2, we collected live leaves of five beech trees randomly chosen in 16 plots per region. Additionally to beech forest, conifer forest (pines at SCH and spruces at HAI and ALB) was added in year 2 (n = 4 plots per Exploratory). For each selected beech tree, leaves of a lower and an upper position within the tree crown were considered, henceforth termed “crown position”. The leaves from heights below 2 meters were sampled with secateurs, whereas leaves from higher crown positions were sampled with a telescopic stick with a clipper at the end controlled by dint of a rope. To ensure that no arthropods escaped due to dropping during branch cutting, we collected those arthropods in a textile funnel positioned underneath the cut branch. The height of the sampled crown positions ranged between 10 cm and 10 m, and depended on the height of the sampled trees and on the accessibility of leaves by the ‘telescopic stick technique’ described above. The size of the sampled trees ranged from less than 1 meter high to fully-grown mature beeches. We estimated the height of the sampled tree (between 20 cm and 30 m) and counted the leaves of one branch per crown position (between 8 and 132 leaves, mean 36), estimated the percentage leaf damage attributable to chewing herbivores of each counted leaf with the aid of sample ‘leaf area loss’ cards (sensu [70]) (S1 Fig) and collected or registered all herbivores, galls and mines on the branch as a measure of herbivore load. In case that we encountered oaks (*Quercus petraea* Liebl. and *Q*. *robur* L.) or sycamore maples (*Acer pseudoplatanus* L.) in the plot, we analyzed herbivory and herbivores of three individuals of these tree species as well (and sampled only three beeches). We conducted the surveys in May when leaf-flushing in *F*. *sylvatica* starts, and during the midseason in July. We started each survey at SCH followed by HAI and ALB, an order that represented expression of tree phenology in the field (J. Mangels personal observation).

Mean values of leaf damage were calculated for each sampled tree per plot and per survey. Mean values were calculated in a similar way of the total number of herbivores, galls and mines per leaf for the surveys in year 2, as an estimator of herbivore density. We additionally calculated the coefficient of variation (CV) of herbivore damage across different trees within a plot, and compared damage patterns in different tree heights (two crown positions differing in height within a tree) based on a vertical stratification index *VS* = *LD*_*U*_ */(LD*_*U*_ *+LD*_*L*_ *)*, where *LD*_*U*_ is the leaf damage assessed at the upper crown position and *LD*_*L*_ the damage at the lower position. For *VS* between 0 and 0.5, herbivore damage is higher in lower parts; values exceeding 0.5 indicate higher damage in the upper crown position. To compare leaf damage experienced by *F*. *sylvatica* with damage of other tree species, we conducted the same assessments of herbivore damage for individuals of *Q*. *robur/petraea* (n = 26) and *A*. *pseudoplatanus* (n = 70).

### Forest management intensity

Defining forest management is a complex issue. The intensity usually varies gradually, which is not well mirrored in simple categorical classifications. To consider different aspects of forest management intensity, we used a combination of two predictor variables: (1) ‘beech dominance’ in the study plots, and (2) ‘harvest intensity’, measured as the proportion of harvested tree volume in the study plots (‘*Iharv*’: [71]). Beech dominance was assessed as the percentage of beech trees (in the shrub and the tree layer) among all tree individuals in the plot area ([72] and Fabrice Grassein, unpublished). The original forests in our study are dominated by beech—a higher proportion of beech trees thus assumedly reflects a lower management intensity. Beech dominance was also negatively related to tree diversity in the studied forest plots (beech dominance vs. Shannon diversity index: *r* = -0.416, *p* < 0.0001). Harvested tree volume in the study plots was quantified by Kahl and Bauhus as the ratio of harvested volume to the sum of standing, harvested and dead wood volume [71]. In accordance with [71], we use the same term *Iharv* for harvested tree volume to address harvest intensity in our analyses.

### Data analysis

Data were analyzed in generalized linear models (commands ‘glm’ for the model) using the statistical software package R 2.15.1 (R Core Team 2012) with the package ‘nlme’ [73]. Leaf damage or herbivore densities were the response variables used in the model, with region (SCH, HAI and ALB), beech dominance and harvest intensity as the three fixed effect terms in a hierarchical order. The significance of the effect terms was tested using Chi^2^-tests (command “anova” based on sequential models of "type I"), and the differences between regions were assessed by Tukey post hoc tests following ANOVA. The data were transformed when necessary (see Table 1) to comply with the assumptions of variance homogeneity (Bartlett test) and normal distribution (Shapiro-Wilk test) of the residuals, and quasi-Poisson distribution was assumed when transformation was unsuccessful. In addition to the deviance and significance level of the GLM factors, we also show the linear regression coefficient for beech dominance and harvest intensity on response variables for a simple interpretation of response directions. To summarize impacts of forest management that may also appear via changes in beech dominance, an additional model for leaf damage was used where beech dominance was removed from the model. To elucidate the interaction effects between region and the continuous predictor variables beech dominance and harvest intensity, we additionally analyzed their effects on the response variables separately per region within the same generalized linear model. Finally, to compare leaf damage across different tree species, we used ANOVA (command ‘aov’) followed by Tukey post hoc tests.

**Table 1:** Effects of region, beech dominance and harvest intensity on leaf damage and on densities of herbivore groups.

		Distribution (transf.)		Region (R)	Beech dominance (B)		Harvest intensity (H)		Interactions
Response			Null-Deviance	Deviance	Deviance	*r*	Deviance	*r*	
Leaf damage	May	n (log+1)	17.50	11.76***	1.07	0.43***	0.52	-0.26** ^a^)	RxB*, BxH***
	July	n (^1/3)	74.87	47.55***	1.35	0.22ns	0.05	-0.04ns ^b^)	
	Nov	n	37	27.34***	0.1	0.1ns	0.44	-0.20ns ^c^)	RxH*
*VS*	May	n	0.87	0.32***	0.08	0.37*	0	-0.1ns	
	July	n	0.74	0.08*	0.18	0.53***	0.02	0.2ns	
CV	May	n (log+1)	1.74	0.09ns	0.09	-0.23ns	0.02	-0.08ns	
	July	n	3.77	0.22ns	0.04	0.12ns	0.30	-0.31*	
	Nov	n (^1/3)	0.08	0.01*	0	0.26ns	0	-0.01ns	
Chewers	May	qp	1.46	0.88***	0	0.02ns	0	-0.14ns	
	July	n (^1/4)	0.01	0.01***	0	-0.16ns	0	0.29*	BxH**
Weevils excl. *R*. *fagi*	May	n (^1/6)	0	0ns	0	-0.07ns	0	-0.13ns	
	July	qp	0.15	0.02**	0	0.07ns	0.06	0.37***	RxH*
*R*. *fagi*	May	qp	0.62	-	0	0.08ns	0.01	-0.13ns	
	July	qp	0.63	-	0	0.34ns	0.01	-0.14ns	
Caterpillars	May	n	0.001	0*	0	0.12ns	0	-0.05ns	
	July	n (sqrt)	0.04	0	0	0.11ns	0	0.23*	RxH***
Aphids	May	qp	2.75	0.1***	0.77	-0.3***	0	-0.1ns	
	July	qp	14.13	4.64***	0.62	-0.22*	0.23	-0.19ns	RxB***, BxH*
Mines	May	qp	10.77	9.33***	0.11	0.2*	0.04	0.13ns	BxH***
	July	n (^1/3)	0.17	0.06***	0	-0.14ns	0	0.12ns	RxBxH*
Galls	May	n (^1/4)	2.64	0.8***	0.28	0.09**	0.10	0.14	
	July	n (^1/3)	3.51	1.81***	0.01	0.15ns	0.01	-0.13ns	RxB***

Generalized mixed model; for the direction of the effects, the linear regression coefficient (*r*) between the residuals of the previous predictors and the respective response variable are shown. *VS* = vertical stratification index, CV = coefficient of variation of leaf damage, *R*. *fagi* = *Rhynchaenus fagi*. Distribution: n (normal) and qp (quasi-Poisson); data transformation for n given in parentheses. Significance levels:. (*p* < 0.1),

* (*p* < 0.05),

** (*p* < 0.01) and

*** (*p* < 0.001).

Degrees of freedom are 1 for beech dominance, 1 for harvest intensity, and 2 for region in May and July (but 1 in November).

Effect of harvest intensity in reduced model without beech dominance:

^a^) *r* = -0.35**;

^b^) *r* = -0.07;

^c^) *r* = -0.22

See S1 Table for complementary analyses of significant interaction effects.

## Results

**Fig 1:**
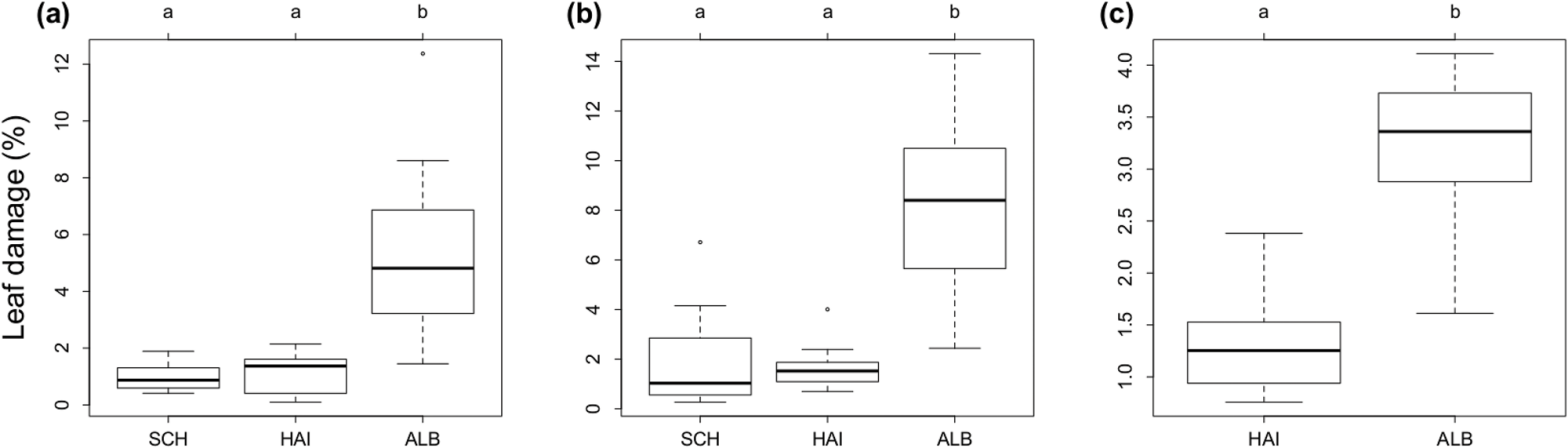
Leaf damage (%) of beech trees in the three study regions. Schorfheide (SCH), Hainich (HAI) and Schwäbische Alb (ALB)—in the surveys in May (a) and July 2013 (b) and November 2012 (c). The letters indicate significant differences between regions (ANOVA, Tukey’s post hoc *p* < 0.05).

**Fig 2:**
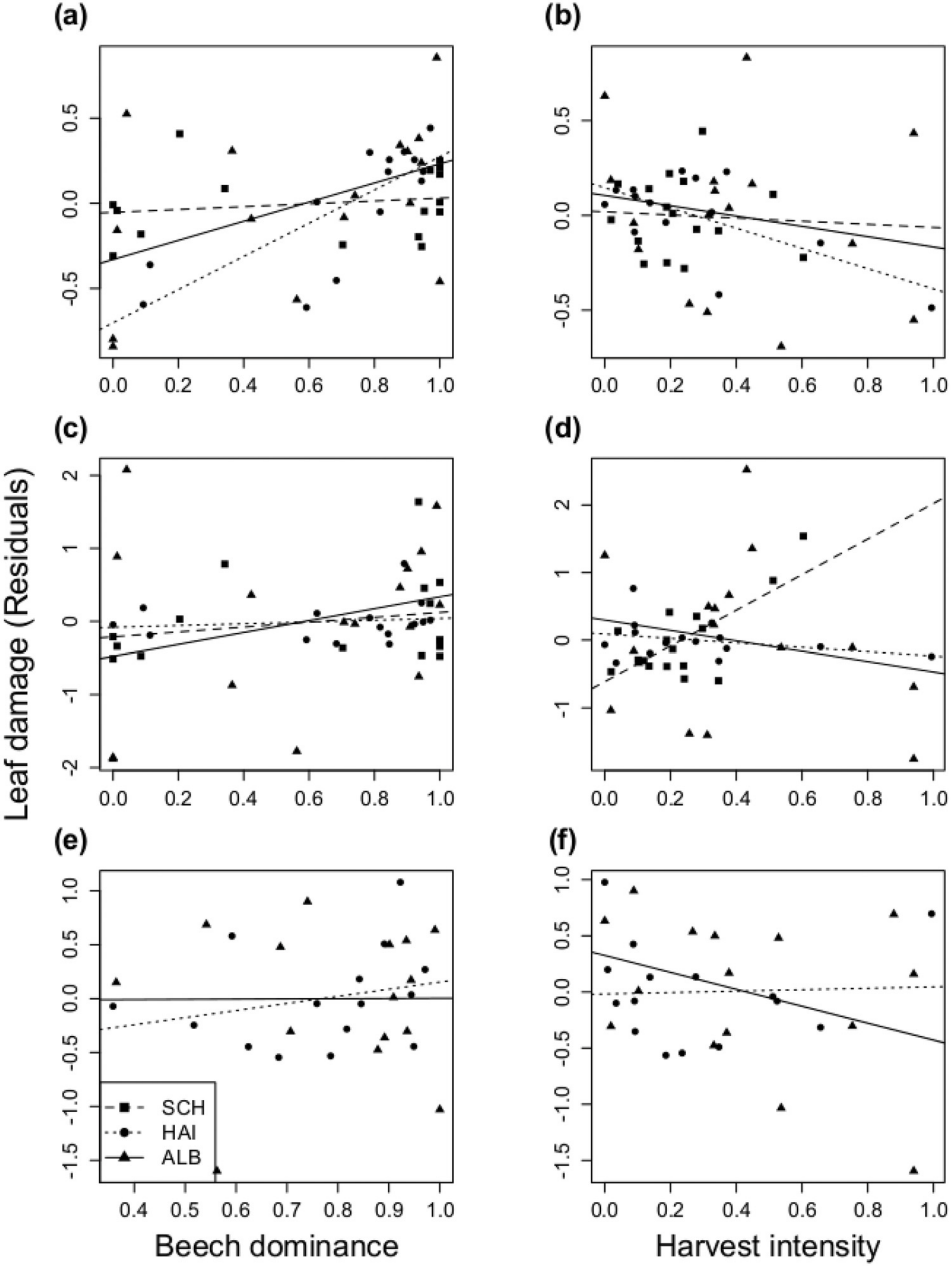
Influence of beech dominance and harvest intensity on leaf damage (residuals) of beech trees. Data were collected in May (a,b), July (c,d) and November (e,f) in the three regions Schorfheide (SCH), Hainich (HAI) and Schwäbische Alb (ALB).

Leaf damage by chewing herbivores differed considerably between regions (Fig 1, Table 1), and both the proportion of *F*. *sylvatica* (beech dominance) and harvest intensity affected the amount of leaf damage and the density of different taxonomic groups of herbivores when the regional differences were accounted for (Fig 2, Table 1). Damage of live leaves was highest in ALB (mean ± SD: 5.3 ± 2.9% in May and 8.1 ± 3.5% in July) and significantly lower in the two other regions (1.0–1.8 ± 0.4–1.9%) (Fig 1, Table 1). Similar results were observed for herbivore damage estimated on fallen leaves, with higher damage in ALB (3.2 ± 0.7%) than in HAI (1.3 ± 0.5%) (Fig 1c, Table 1).

Beech dominance showed a significant positive effect on leaf damage in May, with a significant effect in HAI, a marginally significant trend in ALB and a weak trend in SCH (Fig 2, Table 1, Supporting Information S1 Table). Even after accounting for region and beech dominance, harvest intensity had an additional effect on leaf damage in May, where leaf damage decreased significantly with increasing harvest intensity. The effect of harvesting in May remained significant when beech dominance was not considered as a predictor prior to harvesting in a sequential model (Table 1 footnote). The trends for leaf damage in July and for fallen leaves in November were similar, but not significant. Beech dominance and harvesting were not correlated (Pearson *r* = -0.19, *p* = 0.205).

Region and beech dominance significantly affected the vertical distribution of leaf damage (*VS* index) in May and July (Table 1). Herbivory was most pronounced in upper crown positions in May in ALB (mean *VS* ± SD: 0.61 ± 0.09), but in lower positions in HAI and SCH (0.44 ± 0.14 and 0.42 ± 0.12, respectively). *VS* values were significantly different from 0.5 (which would indicate a similar herbivory in lower and upper crown positions) in ALB (*p* = 0.04) and SCH (*p* = 0.006), but not in HAI (*p* > 0.05). Overall-*VS* values in May were not significantly different from 0.5. In July, the majority of herbivory was found in the upper crown positions (ALB: 0.56 ± 0.14, HAI: 0.57 ± 0.09, SCH: 0.48 ± 0.14), with overall-*VS* values and values from HAI being significantly higher than 0.5 (*p* = 0.04 and 0.003 for overall values and HAI, respectively). These results thus suggest an upward-shift of herbivore feeding in tree crowns during the season at least in HAI and SCH. In forests with high proportions of beech, herbivory was also more pronounced in higher crown positions (Table 1).

The variation of severity of herbivory across trees, expressed by the coefficient of variation (CV) of leaf damage, was not strongly differing between regions (Table 1). The only difference in CV was found between ALB and HAI for data on fallen leaves, where the CV of damage of fallen leaves was significantly lower in ALB (0.25 ± 0.09) than in HAI (0.38 ± 0.20). Beech dominance showed no effect on CV of leaf damage, whereas a weak negative effect (significant only in July) was detected for harvest intensity (Table 1).

Consistent with leaf damage, the abundance of all herbivore guilds—namely chewers, miners, aphids and galls—and of selected taxa, including weevils and lepidopteran caterpillars, significantly differed between regions (Table 1). *Rhynchaenus (Orchestes) fagi* L. was the most abundant weevil and represented 95% of all collected weevil individuals. As this species was detected exclusively in ALB, it was treated separately from the remaining weevils; region was removed as factor in the model. The results suggest that beech dominance and harvest intensity have no significant effect on *R*. *fagi* density. In contrast, beech dominance did have a significant negative effect on the density of aphids (represented by the woolly beech aphid *Phyllaphis fagi* L.) in both surveys, and a significant positive effect on mines and galls in May (Table 1). Harvest intensity had a positive effect on the density of weevils (excluding *R*. *fagi*) and on chewers in July (Table 1). Hence, contrasting responses to harvest intensity were found for leaf damage by chewing herbivores (negative in May) and these herbivore groups (positive) in different months.

A more detailed analysis on the family level of galling arthropods showed a positive effect of beech dominance on gall midges in May (*r* = 0.22; *p* = 0.005) and July (*r* = 0.32; *p* < 0.001) but no effect on gall mites. Analyses on the species level revealed that gall density of the gall mite *Aceria nervisequa* Cane. was positively related to beech dominance in May (*r* = 0.11, *p* = 0.027). Furthermore, beech dominance was positively related to gall density of the gall midges *Mikiola fagi* Hart. (May: *r* = 0.26, *p* = 0.016, July: *r* = 0.42, *p* < 0.001) and *Phegomyia fagicola* Kief. (July: *r* = 0.15, *p* < 0.001). Harvest intensity was positively related to gall density of the gall mite *A*. *nervisequa* in May (*r* = 0.28, *p* = 0.015) and to the gall midge *P*. *fagicola* in May (*r* = 0.25, *p* = 0.028).

Across all sites, average leaf damage and the total density of all leaf-chewing herbivores together were significantly positively correlated in May, but not July (Table 2). This was also confirmed for the caterpillars and for *R*. *fagi* alone, as well as for leaf mines that were not part of the damage assessment (Table 2).

**Table 2:** Relationship between average leaf damage and the total density of herbivore groups.

	May		July	
Predictor	*rs*	*p*	*rs*	*p*
Chewers	0.57	***	0	ns
Weevils excl. *R*. *fagi*	0.11	ns	0	ns
*R*. *fagi*	0.76	***	0.08	ns
Caterpillars	0.31	*	0.02	ns
Aphids	0.13	ns	0.18	ns
Mines	0.79	***	0.03	ns
Galls	-0.04	ns	0.06	ns

Spearman rank correlation; data were obtained in May and July and across all sites (*n* = 48). The correlation between *Rhynchaenus fagi* (*R*. *fagi*) and leaf damage was restricted to the study region ALB (*n* = 16). Significance levels: ns (not significant),

* (*p* < 0.05), and

*** (*p* < 0.001).

*Fagus sylvatica* showed the lowest level of leaf damage compared to the other two studied tree species in July. Highest damage was found for *Acer pseudoplatanus* (mean ± SD: 8.7 ± 11.0), followed by *Quercus* spp. (4.2 ± 4.6%) and *F*. *sylvatica* (3.8 ± 5.2%). The factor tree species influenced leaf damage significantly (*F* = 14.27, *p* < 0.0001). The difference between damage of *F*. *sylvatica* and *A*. *pseudoplatanus* was highly significant (*p* < 0.0001). In May, where *Quercus* had not yet flushed leaves, *A*. *pseudoplatanus* (3.4 ± 6.4%) did not differ significantly from *F*. *sylvatica* (2.5 ± 3.8%).

## Discussion

In our study we investigated the effects of different aspects of forest management on the abundance of arthropod herbivores and on herbivore-related damage to beech trees, the prevailing tree species in natural Central European forests. Forest management was characterized by (1) changes in beech dominance, assuming that a low proportion of beech trees often represents targeted establishment of other timber species, and (2) by harvest intensity. Our results demonstrated that herbivore damage on beech trees may decline (depending on season) with decreasing beech dominance (i.e. a lower herbivory with increasing management intensity) and that it additionally may decline with increasing harvest intensity. The density of leaf chewers and mines was positively related to leaf damage, and several of the studied arthropod groups were found to respond to beech dominance and harvest intensity, albeit in different ways. An analysis of damage patterns in different tree heights indicated a vertical shift of herbivores to higher crown layers during the season and with higher beech dominance.

### Regional differences in herbivore density and leaf damage

The regional differences found for herbivore density and leaf damage are in accordance with other studies showing strong differences in herbivore abundance and impact between study sites differing in climate and other environmental variables [42, 70, 74, 75]. Regional differences in abundance of insect species may be related to abiotic but also biotic environmental parameters, and they may occur for widespread as well as for site-restricted species (for a detailed discussion for German beech forests see [42, 76–78]). Whereas all groups of herbivores varied in abundance across regions in our study, this pattern was particularly obvious for abundance of the beech leaf-miner weevil *R*. *fagi*. This species can be considered as a key herbivore of beech [79–81], and it was the most numerous beetle species in our samples, but was only detected in one of the three regions (ALB). As *R*. *fagi* is known to occur throughout Germany [81], these marked differences in abundance are best explained by fluctuations in population density that are reportedly highly pronounced in this species ([82], and references therein). The importance of this species for herbivore damage in our study was supported by the high correlation of *R*. *fagi* density and overall leaf damage found in May, and the lower herbivore damage in HAI and SCH might be partially explained by the absence of this species.

### Influence of forest management on leaf damage and herbivore density

Despite the strong regional differences in herbivore communities, consistent effects of tree species composition (beech dominance) and harvest intensity on herbivore density and damage were found. This finding emphasizes the potential importance of forest management for interactions between beech trees and their arthropod herbivores in different environmental contexts and it augments information obtained from qualitative herbivory assessments [42]. The increase of damage to beech trees with beech dominance also suggests that some processes described by “*associational resistance hypotheses*” may hold for native Central European beech forests and are not restricted to agricultural [83, 84] or plantation [38, 45, 85] systems that are strongly influenced by land management. Associational resistance can be based on different processes, which may affect herbivores directly via plant traits such as suitability or apparency of focal plants (bottom-up processes), or via indirect effects on the herbivores’ enemies (top-down processes). Different bottom-up processes contribute to associational resistance (see [47, 48] for review of associational resistance hypotheses). As most of these hypotheses have been developed in short-lived agricultural systems—where initial host plant colonization is assumedly more important than in long-lived, perennial systems such as forests—or in young experimental forests, it is still a matter of ongoing investigations how they apply to mature or near-natural forest systems.

An increase of herbivory in pure stands is often explained by the *resource concentration hypothesis* [86, 87]. This hypothesis assumes that herbivores maintain higher densities and success in monospecific stands of their host plants, where presence of many conspecific plants enhances accessibility of host plants for specialist herbivores. In mixed stands, plant diversity may protect host plants by physically [88], visually [89, 90] or chemically [89–91] impeding herbivore access to the host plant. As herbivore species or even stages of the same herbivore species are strongly differing in their foraging behavior and responses to environmental parameters [92, 93], the specific mechanisms underlying an observed damage pattern can only be completely understood when the damaging species have been identified. In our study, this specific identification was obtained for a few species, namely the beech leaf-miner weevil *R*. *fagi*, the woolly beech aphid *P*. *fagi* and a few gall makers. The community of chewing herbivores as a whole responded in a positive way to beech dominance. However, the abundance of the only common chewing herbivore species that can be related to the quantified herbivore damage—the weevil *R*. *fagi*—did not respond to beech dominance in our analysis. The roles of other species need to be elucidated in more detailed studies in beech forests. Nevertheless, possible mechanisms can be deduced from other studies and systems. For example, positive relationships between herbivore damage and host plant dominance may occur in passively dispersing herbivores including early-instar moth larvae, where higher host density increases the probability to land on a suitable host, or for bark beetles, where higher host plant densities allow for buildup of critical population densities [92, 94, 95].

Increasing tree diversity (decreasing host dominance) may also increase herbivore damage, translating to associational susceptibility [37]. This is particularly described for generalist herbivores profiting from mixing different host species, or spilling-over from preferred hosts to less palatable focal plants following exhaustion of the preferred host species [96, 97]. However, associational susceptibility due to increasing tree diversity is not restricted to generalist herbivores, but it can also occur for specialist herbivores avoiding enemies or competitors [45], or those that profit from mixing heterogeneous conspecific plants [98, 99]. In our study, an indication for associational susceptibility was found for infestation of beech by *P*. *fagi*, which was negatively related to beech dominance in spring and summer. In this case, a higher dominance of host trees may dilute the density of early-season fundatrices [100], or it may affect the quality of beech trees as a host. Host quality of trees may depend on tree association, for example as a consequence of facilitation and reduced stress in mixed stands during periods of abiotic stress such as drought [101], which is known to affect the suitability of trees for insect herbivores [102–104], depending on drought intensity[105, 106].

The change in associational effects—from support to suppression of herbivores—by increasing tree diversity (decreasing beech dominance) may also be related to indirect effects of enemies as stated by the *enemies hypothesis* [86, 107]. It is generally assumed that natural enemies of herbivores may profit from increasing plant diversity due to an increasing availability of resource and habitat conditions [84]. In forests, these positive effects of increasing tree diversity have been shown for some predator or parasitoid groups but not for others [38, 65, 108, 109]. To assess whether top-down processes have contributed to effects of beech dominance on herbivores in our study, further investigations on enemy assemblages and on specific herbivores are required.

Besides effects of beech dominance, we also detected an influence of harvest intensity—defined as the proportion of harvested tree volume—on leaf damage and on abundance of some chewing herbivores. Harvesting activities may lead to a simplification of forest structure, which may provoke a reduction of herbivore abundance or species richness [110–112] in different taxonomic groups [14, 113]. These reductions in herbivore occurrence (including both abundance and diversity) may lead to a decrease in leaf consumption, which might explain the observed negative relationship between harvest intensity and herbivore damage in spring and also autumn (see also [114], who found a negative relationship between land use intensity and herbivory in grasslands). The decrease in herbivory might also come along with increased enemy pressure in more strongly disturbed forests, which might be possible for some key enemies [115–117], but is not generally to be expected ([28] and references therein).

### Relationship between herbivore density and damage

We found a generally positive relationship between herbivore density and damage although such a pattern may not always be apparent given the conceptual differences between the two parameters: whereas the assessment of damage integrates over a period of time, the activities of specific herbivores are usually fluctuating over the season and with changes in environmental conditions or resource availability [118]. In our study this shift became apparent by the observed switch of herbivory from lower to higher canopy layers. These shifts may directly track resource availability and quality, but they may also reflect changes in species composition [119]. The shifts in resources and species may also explain why we did not detect effects of beech dominance and harvest intensity on damage later in the season—probably since different effects were masking each other.

## Conclusion

Our study showed that the occurrence of arthropod herbivores in forests and resulting damage to forest trees is influenced by host tree dominance and by differences in harvest intensity. Specifically, herbivore damage to beech trees increased with increasing dominance of beech trees and decreased with increasing harvest intensity. These findings appear to be generalizable at least for European beech forests as they were consistent across forests from three regions varying in biotic and abiotic environmental conditions. At the same time, strong temporal and spatial variation in herbivore occurrence and damage to beech, but also to oak and maple trees, point to the highly conditional nature of herbivory as an ecosystem process.

## Supporting Information

S1 Fig Calculated percentage damage of a fictive beech leaf to support the estimation of damage of sampled leaves. (EPS)

S1 Table Complementary analysis (see Table 1) of significant interaction effects between region, beech dominance and harvest intensity. (DOCX)

S2 Table Data summary. (DOC)
