# Supplementary material for: Tree Species Composition and Harvest Intensity Affect Herbivore Density and Leaf Damage on Beech, Fagus sylvatica, in Different Landscape Contexts
Source: PLoS One. 2015 May 4;10(5):e0126140. doi: 10.1371/journal.pone.0126140 (PMC4418704; doi:10.1371/journal.pone.0126140)
Supplement: S1 Table — (DOCX) [file pone.0126140.s002.docx]

**S1 Table. Complementary analysis (see Table 1) of significant interaction effects between region, beech dominance and harvest intensity**. Generalized mixed model; for the direction of the effects, the linear regression coefficient (*r*) between the residuals of the previous predictors and the respective response variable for the three study regions Schwäbische Alb (ALB), Hainich (HAI) and Schorfheide (SCH) are shown. *R. fagi* = *Rhynchaenus fagi*. Interacting factors: R = region, B = beech dominance, H = harvest intensity. For assessment of interactions between B and H, B is subdivided into two categories B > 0.5 and B < 0.5. Significance levels: . (*p* < 0.1), * (*p* < 0.5), ** (*p* < 0.01) and *** (*p* < 0.001).

| Response | Month | Interaction | *r* ALB | | | *r* HAI | | *r* SCH | | B > 0.5 | | B < 0.5 | |
| --- | --- | --- | --- | --- | --- | --- | --- | --- | --- | --- | --- | --- | --- |
| Leaf damage | May | RxB \| BxH | 0.46 | | . | 0.78 | *** | 0.17 | ns | -0.7 | *** | 0.52 | * |
| Leaf damage | Nov | RxH | -0.35 | | * | 0.04 | ns | - | - | - | - | - | - |
| Chewers | July | BxH | - | | - | - | - | - | - | 0.22 | ns | 0.57 | ** |
| Weevils excl.  *R. fagi* | July | RxH | - | - | | 0.74 | *** | 0.09 | ns | - | - | - | - |
| Caterpillars | July | RxH | -0.28 | | ns | 0.71 | *** | -0.03 | ns | - | - | - | - |
| Aphids | July | RxB \| BxH | -0.52 | | ** | -0.08 | ns | 0.22 | ns | -0.55 | ** | -0.18 | ns |
| Mines | May | BxH ^a)^ | - | | - | - | - | - | - | -0.39 | ** | 0.59 | *** |
| Galls | July | RxB | -0.6 | | ** | -0.29 | ns | 0.59 | ** | - | - | - | - |
| ^a)^ RxBxH: In July, effects of beech dominance and harvest intensity on mines in SCH were not significantly different, whereas the effect of beech dominance was significantly negative in HAI (Pearson *r* = -0.55, *p* = 0.016). The analysis in ALB showed a significant interaction effect between beech dominance and harvest intensity (interaction effect: *p* = 0.049; beech dominance <0.5: *r* = 0.92, *p* < 0.001; beech dominance > 0.5: *r* = -0.17, *p* = 0.62). | | | | | | | | | | | | | |
