# Supplementary material for: Tree Species Composition and Harvest Intensity Affect Herbivore Density and Leaf Damage on Beech, Fagus sylvatica, in Different Landscape Contexts
Source: PLoS One. 2015 May 4;10(5):e0126140. doi: 10.1371/journal.pone.0126140 (PMC4418704; doi:10.1371/journal.pone.0126140)
Supplement: S2 Table — (DOC) [file pone.0126140.s003.doc]

**S2 Table. Data summary.** Description of variables is followed by data underlying analyses presented in manuscript.

| **Variable** | **Description** |
| --- | --- |
| Region | Alb, Schorfheide, Hainich |
| Plot | Exploratory plot: AEW = ALB, HEW = HAI, SEW = SCH |
| Month | Study months: May, July, November |
| Beech dominance | Percentage of beech trees among all tree individuals in the plot area |
| Harvest intensity | *Iharv;*  See: Kahl T and Bauhus J (2014) An index of forest management intensity based on assessment of harvested tree volume, tree species composition and dead wood origin. Nat Conserv 7: 15-27. doi:10.3897/natureconservation.7.7281.  Data on *Iharv* obtained from: Tiemo Kahl, Univ Freiburg, Fac Environm & Nat Resources, Chair Silviculture, Tennenbacherstr 4, D-79106 Freiburg, Germany. tiemo.kahl@waldbau.uni-freiburg.de. |
| Leaf damage | Percent missing leaf area |
| *R. fagi* | *Rhynchaenus fagi* density per leaf |
| Weevils | All weevil density per leaf |
| Weevils_excl_*R. fagi* | Weevil density per leaf without *Rhynchaenus fagi* |
| Caterpillars | Caterpillar density per leaf |
| Galls | Gall density per leaf |
| Aphids | Aphid density per leaf |
| Mines | Mine density per leaf |
| Chewers | Chewer density per leaf; Chewers = sum caterpillars, weevils |
| Gall midges | Gall midge density per leaf |
| Gall mites | Gall mite density per leaf |
| *A. stenaspis* | *Acalitus stenaspis* density per leaf |
| *A. nervisequa* | *Aceria nervisequa* density per leaf |
| *H. annulipes* | *Hartigiola annulipes* density per leaf |
| *M. fagi* | *Mikiola fagi* density per leaf |
| *P. fagicola* | *Phegomyia fagicola* density per leaf |

| **Region** | **Plot** | **Month** | **Beech**  **dominance** | **Leaf**  **damage** | **Harvest**  **intensity** | ***R.***  ***fagi*** | **Weevils** | **Weevils**  **excl_*R. fagi*** | **Caterpillars** | **Galls** | **Aphids** |
| --- | --- | --- | --- | --- | --- | --- | --- | --- | --- | --- | --- |
| **ALB** | **AEW1** | **May** | 0 | 1.451 | 0.312 | 0 | 0 | 0 | 0.003 | 0 | 0.142 |
| **ALB** | **AEW2** | **May** | 0.042 | 8.606 | 0.432 | 0.124 | 0.124 | 0 | 0.003 | 0 | 0.295 |
| **ALB** | **AEW3** | **May** | 0.013 | 3.853 | 0.448 | 0.007 | 0.011 | 0.004 | 0 | 0.004 | 0 |
| **ALB** | **AEW4** | **May** | 0.707 | 4.230 | 0.753 | 0.005 | 0.005 | 0 | 0.002 | 0.040 | 0 |
| **ALB** | **AEW5** | **May** | 0.944 | 6.214 | 0.378 | 0.063 | 0.063 | 0 | 0.008 | 0 | 0.008 |
| **ALB** | **AEW6** | **May** | 0.901 | 6.709 | 0.335 | 0.063 | 0.063 | 0 | 0.010 | 0.033 | 0.033 |
| **ALB** | **AEW7** | **May** | 0.878 | 6.996 | 0.332 | 0.007 | 0.019 | 0.012 | 0.002 | 0 | 0 |
| **ALB** | **AEW8** | **May** | 0.990 | 12.370 | 0 | 0.025 | 0.027 | 0.002 | 0.002 | 0 | 0 |
| **ALB** | **AEW9** | **May** | 0.740 | 4.943 | 0.088 | 0.059 | 0.059 | 0 | 0.004 | 0 | 0 |
| **ALB** | **AEW11** | **May** | 0 | 1.560 | 0.257 | 0 | 0.045 | 0.045 | 0 | 0 | 0 |
| **ALB** | **AEW17** | **May** | 1.000 | 2.588 | 0.537 | 0.020 | 0.027 | 0.007 | 0.007 | 0.067 | 0 |
| **ALB** | **AEW18** | **May** | 0.936 | 7.319 | 0.019 | 0.094 | 0.109 | 0.015 | 0.007 | 0 | 0.005 |
| **ALB** | **AEW25** | **May** | 0.563 | 2.227 | 0.94 | 0 | 0 | 0 | 0.005 | 0 | 0 |
| **ALB** | **AEW27** | **May** | 0.364 | 6.733 | 0.94 | 0.065 | 0.070 | 0.005 | 0.005 | 0 | 0 |
| **ALB** | **AEW30** | **May** | 0.423 | 4.183 | 0.316 | 0.030 | 0.030 | 0 | 0.003 | 0 | 0 |
| **ALB** | **AEW49** | **May** | 0.909 | 4.688 | 0.103 | 0.011 | 0.011 | 0 | 0.003 | 0 | 0 |
| **HAI** | **HEW1** | **May** | 0.113 | 0.408 | 0.371 | 0 | 0 | 0 | 0.004 | 0 | 0 |
| **HAI** | **HEW3** | **May** | 0.093 | 0.115 | 0.326 | 0 | 0.002 | 0.002 | 0 | 0.262 | 0.002 |
| **HAI** | **HEW4** | **May** | 0.592 | 0.096 | 0.994 | 0 | 0 | 0 | 0 | 0.015 | 0 |
| **HAI** | **HEW5** | **May** | 0.684 | 0.253 | 0.347 | 0 | 0 | 0 | 0 | 0.031 | 0 |
| **HAI** | **HEW6** | **May** | 0.944 | 1.303 | 0.091 | 0 | 0 | 0 | 0.002 | 0.025 | 0 |
| **HAI** | **HEW10** | **May** | 0.846 | 1.542 | 0.035 | 0 | 0 | 0 | 0 | 0.072 | 0 |
| **HAI** | **HEW11** | **May** | 0.891 | 1.392 | 0.087 | 0 | 0 | 0 | 0.002 | 0.100 | 0.002 |
| **HAI** | **HEW12** | **May** | 0.923 | 1.147 | 0 | 0 | 0 | 0 | 0.009 | 0.030 | 0 |
| **HAI** | **HEW16** | **May** | 0.624 | 1.037 | 0.092 | 0 | 0 | 0 | 0 | 0.003 | 0 |
| **HAI** | **HEW17** | **May** | 0.817 | 0.922 | 0.656 | 0 | 0 | 0 | 0.003 | 0.361 | 0 |
| **HAI** | **HEW21** | **May** | 0.971 | 2.605 | 0.277 | 0 | 0.005 | 0.005 | 0 | 0.049 | 0 |
| **HAI** | **HEW22** | **May** | 0.949 | 1.436 | 0.186 | 0 | 0.003 | 0.003 | 0.007 | 0.244 | 0 |
| **HAI** | **HEW36** | **May** | 0.842 | 1.744 | 0.138 | 0 | 0 | 0 | 0.004 | 0 | 0 |
| **HAI** | **HEW47** | **May** | 0.786 | 1.724 | 0.235 | 0 | 0 | 0 | 0.006 | 0.045 | 0 |
| **SCH** | **SEW1** | **May** | 0.012 | 0.843 | 0.207 | 0 | 0 | 0 | 0.013 | 0 | 0 |
| **SCH** | **SEW2** | **May** | 0 | 0.905 | 0.188 | 0 | 0 | 0 | 0.008 | 0 | 0 |
| **SCH** | **SEW3** | **May** | 0 | 0.411 | 0.119 | 0 | 0 | 0 | 0.002 | 0 | 0 |
| **SCH** | **SEW4** | **May** | 0.703 | 0.507 | 0.189 | 0 | 0 | 0 | 0.002 | 0.038 | 0 |
| **SCH** | **SEW5** | **May** | 0.944 | 0.492 | 0.242 | 0 | 0 | 0 | 0.003 | 0.005 | 0 |
| **SCH** | **SEW6** | **May** | 0.935 | 0.579 | 0.604 | 0 | 0 | 0 | 0.005 | 0.021 | 0 |
| **SCH** | **SEW7** | **May** | 1.000 | 1.043 | 0.019 | 0 | 0 | 0 | 0.008 | 0.004 | 0 |
| **SCH** | **SEW8** | **May** | 0.970 | 1.334 | 0.04 | 0 | 0 | 0 | 0.007 | 0.094 | 0 |
| **SCH** | **SEW9** | **May** | 1.000 | 0.828 | 0.346 | 0 | 0.002 | 0.002 | 0.002 | 0 | 0 |
| **SCH** | **SEW18** | **May** | 0.085 | 0.604 | 0.102 | 0 | 0 | 0 | 0.006 | 0 | 0 |
| **SCH** | **SEW35** | **May** | 1.000 | 1.471 | 0.197 | 0 | 0 | 0 | 0.006 | 0.021 | 0 |
| **SCH** | **SEW36** | **May** | 1.000 | 1.371 | 0.24 | 0 | 0.006 | 0.006 | 0 | 0.076 | 0 |
| **SCH** | **SEW37** | **May** | 1.000 | 1.280 | 0.135 | 0 | 0 | 0 | 0.012 | 0.025 | 0 |
| **SCH** | **SEW41** | **May** | 0.342 | 1.096 | 0.512 | 0 | 0 | 0 | 0.002 | 0.010 | 0 |
| **SCH** | **SEW43** | **May** | 0.952 | 0.835 | 0.279 | 0 | 0 | 0 | 0.008 | 0.011 | 0 |
| **SCH** | **SEW48** | **May** | 0.204 | 1.891 | 0.297 | 0 | 0 | 0 | 0.011 | 0.004 | 0 |
| **ALB** | **AEW1** | **July** | 0 | 2.437 | 0.312 | 0.049 | 0.049 | 0 | 0.003 | 0.046 | 0 |
| **ALB** | **AEW2** | **July** | 0.042 | 14.314 | 0.432 | 0.262 | 0.262 | 0 | 0 | 0 | 1.313 |
| **ALB** | **AEW3** | **July** | 0.013 | 10.743 | 0.448 | 0.040 | 0.040 | 0 | 0 | 0.029 | 0.072 |
| **ALB** | **AEW4** | **July** | 0.707 | 8.046 | 0.753 | 0.040 | 0.040 | 0 | 0 | 0.008 | 0 |
| **ALB** | **AEW5** | **July** | 0.944 | 10.944 | 0.378 | 0.114 | 0.114 | 0 | 0 | 0 | 0.017 |
| **ALB** | **AEW6** | **July** | 0.901 | 10.245 | 0.335 | 0.108 | 0.108 | 0 | 0 | 0 | 0 |
| **ALB** | **AEW7** | **July** | 0.878 | 9.471 | 0.332 | 0.019 | 0.019 | 0 | 0.002 | 0 | 0.056 |
| **ALB** | **AEW8** | **July** | 0.990 | 12.826 | 0 | 0.110 | 0.110 | 0 | 0.002 | 0.017 | 0.119 |
| **ALB** | **AEW9** | **July** | 0.740 | 7.975 | 0.088 | 0.002 | 0.002 | 0 | 0 | 0 | 0.135 |
| **ALB** | **AEW11** | **July** | 0 | 2.498 | 0.257 | 0.146 | 0.146 | 0 | 0 | 0.099 | 1.016 |
| **ALB** | **AEW17** | **July** | 1.000 | 8.757 | 0.537 | 0.106 | 0.106 | 0 | 0 | 0 | 0.021 |
| **ALB** | **AEW18** | **July** | 0.936 | 5.826 | 0.019 | 0.078 | 0.078 | 0 | 0 | 0 | 0.189 |
| **ALB** | **AEW25** | **July** | 0.563 | 2.753 | 0.94 | 0.107 | 0.107 | 0 | 0 | 0.029 | 0 |
| **ALB** | **AEW27** | **July** | 0.364 | 5.465 | 0.94 | 0.353 | 0.353 | 0 | 0 | 0 | 0 |
| **ALB** | **AEW30** | **July** | 0.423 | 9.164 | 0.316 | 0.060 | 0.060 | 0 | 0 | 0 | 0.030 |
| **ALB** | **AEW49** | **July** | 0.909 | 7.860 | 0.103 | 0.071 | 0.071 | 0 | 0 | 0 | 0.055 |
| **HAI** | **HEW1** | **July** | 0.113 | 1.064 | 0.371 | 0 | 0 | 0 | 0 | 0.447 | 0 |
| **HAI** | **HEW3** | **July** | 0.093 | 2.187 | 0.326 | 0 | 0 | 0 | 0.004 | 0.247 | 0.012 |
| **HAI** | **HEW4** | **July** | 0.592 | 0.875 | 0.994 | 0 | 0.014 | 0.014 | 0.014 | 0 | 0 |
| **HAI** | **HEW5** | **July** | 0.684 | 0.713 | 0.347 | 0 | 0 | 0 | 0.008 | 0.112 | 0.032 |
| **HAI** | **HEW6** | **July** | 0.944 | 2.391 | 0.091 | 0 | 0 | 0 | 0 | 0.377 | 0.006 |
| **HAI** | **HEW10** | **July** | 0.846 | 0.694 | 0.035 | 0 | 0 | 0 | 0 | 0.068 | 0.048 |
| **HAI** | **HEW11** | **July** | 0.891 | 4.006 | 0.087 | 0 | 0 | 0 | 0.003 | 0.142 | 0.077 |
| **HAI** | **HEW12** | **July** | 0.923 | 1.519 | 0 | 0 | 0 | 0 | 0 | 0.094 | 0.014 |
| **HAI** | **HEW13** | **July** | 0 | 1.490 | 0.35 | 0 | 0 | 0 | 0 | 0.559 | 0.062 |
| **HAI** | **HEW16** | **July** | 0.624 | 1.961 | 0.092 | 0 | 0 | 0 | 0 | 0.030 | 0.027 |
| **HAI** | **HEW17** | **July** | 0.817 | 1.393 | 0.656 | 0 | 0 | 0 | 0.003 | 0.095 | 0 |
| **HAI** | **HEW21** | **July** | 0.971 | 1.679 | 0.277 | 0 | 0 | 0 | 0.003 | 0.314 | 0.003 |
| **HAI** | **HEW22** | **July** | 0.949 | 1.604 | 0.186 | 0 | 0 | 0 | 0 | 0.644 | 0.034 |
| **HAI** | **HEW36** | **July** | 0.842 | 1.113 | 0.138 | 0 | 0 | 0 | 0 | 0.124 | 0 |
| **HAI** | **HEW47** | **July** | 0.786 | 1.777 | 0.235 | 0.009 | 0.009 | 0 | 0 | 0.247 | 0 |
| **SCH** | **SEW1** | **July** | 0.012 | 0.783 | 0.207 | 0 | 0 | 0 | 0 | 0.018 | 0.465 |
| **SCH** | **SEW2** | **July** | 0 | 1.166 | 0.188 | 0 | 0 | 0 | 0 | 0.021 | 0.053 |
| **SCH** | **SEW3** | **July** | 0 | 0.262 | 0.119 | 0 | 0 | 0 | 0 | 0.087 | 0.135 |
| **SCH** | **SEW4** | **July** | 0.703 | 0.462 | 0.189 | 0 | 0 | 0 | 0 | 0.036 | 0.122 |
| **SCH** | **SEW5** | **July** | 0.944 | 0.398 | 0.242 | 0 | 0 | 0 | 0.003 | 0.228 | 0.228 |
| **SCH** | **SEW6** | **July** | 0.935 | 7.496 | 0.604 | 0 | 0 | 0 | 0 | 0.127 | 0.178 |
| **SCH** | **SEW7** | **July** | 1.000 | 0.773 | 0.019 | 0 | 0 | 0 | 0 | 0.283 | 0.260 |
| **SCH** | **SEW8** | **July** | 0.970 | 2.533 | 0.04 | 0 | 0 | 0 | 0 | 0.063 | 0.385 |
| **SCH** | **SEW9** | **July** | 1.000 | 0.369 | 0.346 | 0 | 0 | 0 | 0 | 0.166 | 0.197 |
| **SCH** | **SEW18** | **July** | 0.085 | 0.379 | 0.102 | 0 | 0 | 0 | 0 | 0.006 | 0.231 |
| **SCH** | **SEW35** | **July** | 1.000 | 3.400 | 0.197 | 0 | 0 | 0 | 0 | 0.330 | 0.741 |
| **SCH** | **SEW36** | **July** | 1.000 | 1.032 | 0.24 | 0 | 0.003 | 0.003 | 0 | 0.823 | 0.363 |
| **SCH** | **SEW37** | **July** | 1.000 | 1.019 | 0.135 | 0 | 0 | 0 | 0.003 | 0.388 | 0.353 |
| **SCH** | **SEW41** | **July** | 0.342 | 4.153 | 0.512 | 0 | 0 | 0 | 0 | 0.090 | 0.540 |
| **SCH** | **SEW43** | **July** | 0.952 | 3.166 | 0.279 | 0 | 0.009 | 0.009 | 0.009 | 0.342 | 0.377 |
| **SCH** | **SEW48** | **July** | 0.204 | 1.886 | 0.297 | 0 | 0 | 0 | 0 | 0.117 | 0.310 |
| **ALB** | **AEW4** | **November** | 0.707 | 2.906 | 0.753 | na | na | na | na | na | na |
| **ALB** | **AEW5** | **November** | 0.944 | 3.383 | 0.378 | na | na | na | na | na | na |
| **ALB** | **AEW6** | **November** | 0.901 | 3.303 | 0.335 | na | na | na | na | na | na |
| **ALB** | **AEW7** | **November** | 0.878 | 2.735 | 0.332 | na | na | na | na | na | na |
| **ALB** | **AEW8** | **November** | 0.990 | 3.847 | 0 | na | na | na | na | na | na |
| **ALB** | **AEW9** | **November** | 0.740 | 4.110 | 0.088 | na | na | na | na | na | na |
| **ALB** | **AEW17** | **November** | 1.000 | 2.182 | 0.537 | na | na | na | na | na | na |
| **ALB** | **AEW18** | **November** | 0.936 | 2.908 | 0.019 | na | na | na | na | na | na |
| **ALB** | **AEW25** | **November** | 0.563 | 1.612 | 0.94 | na | na | na | na | na | na |
| **ALB** | **AEW26** | **November** | 0.542 | 3.897 | 0.879 | na | na | na | na | na | na |
| **ALB** | **AEW27** | **November** | 0.364 | 3.361 | 0.94 | na | na | na | na | na | na |
| **ALB** | **AEW41** | **November** | 0.892 | 2.849 | 0.371 | na | na | na | na | na | na |
| **ALB** | **AEW42** | **November** | 0.687 | 3.689 | 0.529 | na | na | na | na | na | na |
| **ALB** | **AEW49** | **November** | 0.909 | 3.222 | 0.103 | na | na | na | na | na | na |
| **ALB** | **AEW50** | **November** | 0.934 | 3.750 | 0.268 | na | na | na | na | na | na |
| **HAI** | **HEW4** | **November** | 0.592 | 1.883 | 0.994 | na | na | na | na | na | na |
| **HAI** | **HEW5** | **November** | 0.684 | 0.757 | 0.347 | na | na | na | na | na | na |
| **HAI** | **HEW6** | **November** | 0.944 | 1.338 | 0.091 | na | na | na | na | na | na |
| **HAI** | **HEW10** | **November** | 0.846 | 1.254 | 0.035 | na | na | na | na | na | na |
| **HAI** | **HEW11** | **November** | 0.891 | 1.810 | 0.087 | na | na | na | na | na | na |
| **HAI** | **HEW12** | **November** | 0.923 | 2.381 | 0 | na | na | na | na | na | na |
| **HAI** | **HEW16** | **November** | 0.624 | 0.855 | 0.092 | na | na | na | na | na | na |
| **HAI** | **HEW17** | **November** | 0.817 | 1.020 | 0.656 | na | na | na | na | na | na |
| **HAI** | **HEW18** | **November** | 0.759 | 1.256 | 0.511 | na | na | na | na | na | na |
| **HAI** | **HEW21** | **November** | 0.971 | 1.572 | 0.277 | na | na | na | na | na | na |
| **HAI** | **HEW22** | **November** | 0.949 | 0.858 | 0.186 | na | na | na | na | na | na |
| **HAI** | **HEW23** | **November** | 0.517 | 1.056 | 0.524 | na | na | na | na | na | na |
| **HAI** | **HEW36** | **November** | 0.842 | 1.484 | 0.138 | na | na | na | na | na | na |
| **HAI** | **HEW40** | **November** | 0.358 | 1.231 | 0.009 | na | na | na | na | na | na |
| **HAI** | **HEW47** | **November** | 0.786 | 0.770 | 0.235 | na | na | na | na | na | na |

| **Region** | **Plot** | **Month** | **Mines** | **Chewers** | **Gall**  **midges** | **Gall**  **mites** | ***A.***  ***stenaspis*** | ***A.***  ***nervisequa*** | ***H.***  ***annulipes*** | ***M.***  ***fagi*** | ***P.***  ***fagicola*** |
| --- | --- | --- | --- | --- | --- | --- | --- | --- | --- | --- | --- |
| **ALB** | **AEW1** | **May** | 0.214 | 0.003 | 0 | 0 | 0 | 0 | 0 | 0 | 0 |
| **ALB** | **AEW2** | **May** | 0.402 | 0.127 | 0 | 0 | 0 | 0 | 0 | 0 | 0 |
| **ALB** | **AEW3** | **May** | 0.336 | 0.011 | 0.004 | 0 | 0 | 0 | 0 | 0.004 | 0 |
| **ALB** | **AEW4** | **May** | 0.225 | 0.007 | 0 | 0.040 | 0.040 | 0 | 0 | 0 | 0 |
| **ALB** | **AEW5** | **May** | 0.523 | 0.071 | 0 | 0.042 | 0 | 0.042 | 0 | 0 | 0 |
| **ALB** | **AEW6** | **May** | 0.370 | 0.073 | 0 | 0 | 0 | 0 | 0 | 0 | 0 |
| **ALB** | **AEW7** | **May** | 0.462 | 0.022 | 0 | 0 | 0 | 0 | 0 | 0 | 0 |
| **ALB** | **AEW8** | **May** | 0.338 | 0.028 | 0 | 0 | 0 | 0 | 0 | 0 | 0 |
| **ALB** | **AEW9** | **May** | 0.345 | 0.063 | 0 | 0 | 0 | 0 | 0 | 0 | 0 |
| **ALB** | **AEW11** | **May** | 0.015 | 0.045 | 0 | 0 | 0 | 0 | 0 | 0 | 0 |
| **ALB** | **AEW17** | **May** | 0.258 | 0.034 | 0.003 | 0.064 | 0 | 0.064 | 0 | 0.003 | 0 |
| **ALB** | **AEW18** | **May** | 0.430 | 0.116 | 0 | 0 | 0 | 0 | 0 | 0 | 0 |
| **ALB** | **AEW25** | **May** | 0.207 | 0.005 | 0 | 0 | 0 | 0 | 0 | 0 | 0 |
| **ALB** | **AEW27** | **May** | 0.664 | 0.075 | 0 | 0 | 0 | 0 | 0 | 0 | 0 |
| **ALB** | **AEW30** | **May** | 0.213 | 0.034 | 0 | 0 | 0 | 0 | 0 | 0 | 0 |
| **ALB** | **AEW49** | **May** | 0.279 | 0.013 | 0 | 0 | 0 | 0 | 0 | 0 | 0 |
| **HAI** | **HEW1** | **May** | 0 | 0.004 | 0 | 0 | 0 | 0 | 0 | 0 | 0 |
| **HAI** | **HEW3** | **May** | 0.009 | 0.002 | 0.026 | 0.024 | 0 | 0.024 | 0 | 0.021 | 0.005 |
| **HAI** | **HEW4** | **May** | 0.012 | 0 | 0.015 | 0 | 0 | 0 | 0 | 0.015 | 0 |
| **HAI** | **HEW5** | **May** | 0.002 | 0 | 0.031 | 0 | 0 | 0 | 0 | 0.031 | 0 |
| **HAI** | **HEW6** | **May** | 0.002 | 0.002 | 0.025 | 0 | 0 | 0 | 0 | 0.025 | 0 |
| **HAI** | **HEW10** | **May** | 0.049 | 0 | 0.072 | 0 | 0 | 0 | 0 | 0.066 | 0.006 |
| **HAI** | **HEW11** | **May** | 0.044 | 0.002 | 0.012 | 0.087 | 0.087 | 0 | 0 | 0.012 | 0 |
| **HAI** | **HEW12** | **May** | 0.009 | 0.009 | 0.027 | 0.003 | 0 | 0.003 | 0 | 0.027 | 0 |
| **HAI** | **HEW16** | **May** | 0.050 | 0 | 0.003 | 0 | 0 | 0 | 0 | 0.003 | 0 |
| **HAI** | **HEW17** | **May** | 0.023 | 0.003 | 0.330 | 0.031 | 0 | 0.031 | 0 | 0.039 | 0.291 |
| **HAI** | **HEW21** | **May** | 0.052 | 0.005 | 0.023 | 0 | 0 | 0 | 0 | 0 | 0.023 |
| **HAI** | **HEW22** | **May** | 0.027 | 0.010 | 0.244 | 0 | 0 | 0 | 0 | 0.014 | 0.230 |
| **HAI** | **HEW36** | **May** | 0.047 | 0.004 | 0 | 0 | 0 | 0 | 0 | 0 | 0 |
| **HAI** | **HEW47** | **May** | 0.031 | 0.006 | 0 | 0.045 | 0 | 0.045 | 0 | 0 | 0 |
| **SCH** | **SEW1** | **May** | 0 | 0.013 | 0 | 0 | 0 | 0 | 0 | 0 | 0 |
| **SCH** | **SEW2** | **May** | 0 | 0.008 | 0 | 0 | 0 | 0 | 0 | 0 | 0 |
| **SCH** | **SEW3** | **May** | 0 | 0.002 | 0 | 0 | 0 | 0 | 0 | 0 | 0 |
| **SCH** | **SEW4** | **May** | 0 | 0.002 | 0.038 | 0 | 0 | 0 | 0 | 0.038 | 0 |
| **SCH** | **SEW5** | **May** | 0 | 0.003 | 0.005 | 0 | 0 | 0 | 0 | 0.005 | 0 |
| **SCH** | **SEW6** | **May** | 0 | 0.005 | 0.021 | 0 | 0 | 0 | 0 | 0.021 | 0 |
| **SCH** | **SEW7** | **May** | 0 | 0.008 | 0.004 | 0 | 0 | 0 | 0 | 0.004 | 0 |
| **SCH** | **SEW8** | **May** | 0 | 0.007 | 0.094 | 0 | 0 | 0 | 0 | 0.094 | 0 |
| **SCH** | **SEW9** | **May** | 0 | 0.003 | 0 | 0 | 0 | 0 | 0 | 0 | 0 |
| **SCH** | **SEW18** | **May** | 0 | 0.006 | 0 | 0 | 0 | 0 | 0 | 0 | 0 |
| **SCH** | **SEW35** | **May** | 0 | 0.006 | 0.021 | 0 | 0 | 0 | 0 | 0.021 | 0 |
| **SCH** | **SEW36** | **May** | 0 | 0.006 | 0 | 0.076 | 0.076 | 0 | 0 | 0 | 0 |
| **SCH** | **SEW37** | **May** | 0 | 0.012 | 0.025 | 0 | 0 | 0 | 0 | 0.025 | 0 |
| **SCH** | **SEW41** | **May** | 0 | 0.002 | 0.007 | 0.002 | 0 | 0.002 | 0 | 0.007 | 0 |
| **SCH** | **SEW43** | **May** | 0 | 0.008 | 0.011 | 0 | 0 | 0 | 0 | 0.011 | 0 |
| **SCH** | **SEW48** | **May** | 0 | 0.011 | 0.004 | 0 | 0 | 0 | 0 | 0.004 | 0 |
| **ALB** | **AEW1** | **July** | 0.219 | 0.052 | 0 | 0.046 | 0.018 | 0.027 | 0 | 0 | 0 |
| **ALB** | **AEW2** | **July** | 0.548 | 0.262 | 0 | 0 | 0 | 0 | 0 | 0 | 0 |
| **ALB** | **AEW3** | **July** | 0.272 | 0.040 | 0.029 | 0 | 0 | 0 | 0.029 | 0 | 0 |
| **ALB** | **AEW4** | **July** | 0.259 | 0.040 | 0.008 | 0 | 0 | 0 | 0.008 | 0 | 0 |
| **ALB** | **AEW5** | **July** | 0.646 | 0.114 | 0 | 0 | 0 | 0 | 0 | 0 | 0 |
| **ALB** | **AEW6** | **July** | 0.395 | 0.108 | 0 | 0 | 0 | 0 | 0 | 0 | 0 |
| **ALB** | **AEW7** | **July** | 0.302 | 0.022 | 0 | 0 | 0 | 0 | 0 | 0 | 0 |
| **ALB** | **AEW8** | **July** | 0.310 | 0.112 | 0 | 0.017 | 0.017 | 0 | 0 | 0 | 0 |
| **ALB** | **AEW9** | **July** | 0.560 | 0.002 | 0 | 0 | 0 | 0 | 0 | 0 | 0 |
| **ALB** | **AEW11** | **July** | 0.042 | 0.146 | 0 | 0.125 | 0 | 0.125 | 0 | 0 | 0 |
| **ALB** | **AEW17** | **July** | 0.312 | 0.106 | 0 | 0 | 0 | 0 | 0 | 0 | 0 |
| **ALB** | **AEW18** | **July** | 0.256 | 0.078 | 0 | 0 | 0 | 0 | 0 | 0 | 0 |
| **ALB** | **AEW25** | **July** | 0.278 | 0.107 | 0 | 0.029 | 0.029 | 0 | 0 | 0 | 0 |
| **ALB** | **AEW27** | **July** | 0.856 | 0.353 | 0 | 0 | 0 | 0 | 0 | 0 | 0 |
| **ALB** | **AEW30** | **July** | 0.199 | 0.060 | 0 | 0 | 0 | 0 | 0 | 0 | 0 |
| **ALB** | **AEW49** | **July** | 0.265 | 0.071 | 0 | 0 | 0 | 0 | 0 | 0 | 0 |
| **HAI** | **HEW1** | **July** | 0.080 | 0 | 0 | 0.447 | 0 | 0.447 | 0 | 0 | 0 |
| **HAI** | **HEW3** | **July** | 0.171 | 0.004 | 0.040 | 0.207 | 0.052 | 0.155 | 0.016 | 0.024 | 0 |
| **HAI** | **HEW4** | **July** | 0.065 | 0.029 | 0 | 0 | 0 | 0 | 0 | 0 | 0 |
| **HAI** | **HEW5** | **July** | 0.056 | 0.008 | 0.044 | 0.068 | 0 | 0.068 | 0 | 0.044 | 0 |
| **HAI** | **HEW6** | **July** | 0.081 | 0 | 0.032 | 0.345 | 0 | 0.345 | 0 | 0.032 | 0 |
| **HAI** | **HEW10** | **July** | 0.065 | 0 | 0.058 | 0.010 | 0 | 0.010 | 0.014 | 0.044 | 0 |
| **HAI** | **HEW11** | **July** | 0.071 | 0.003 | 0.074 | 0.068 | 0 | 0.068 | 0.009 | 0.065 | 0 |
| **HAI** | **HEW12** | **July** | 0.083 | 0 | 0.028 | 0.066 | 0 | 0.066 | 0 | 0.023 | 0.006 |
| **HAI** | **HEW13** | **July** | 0.148 | 0 | 0.037 | 0.522 | 0 | 0.522 | 0.019 | 0.019 | 0 |
| **HAI** | **HEW16** | **July** | 0.116 | 0 | 0 | 0.030 | 0 | 0.030 | 0 | 0 | 0 |
| **HAI** | **HEW17** | **July** | 0.092 | 0.003 | 0.003 | 0.092 | 0.010 | 0.082 | 0 | 0.003 | 0 |
| **HAI** | **HEW21** | **July** | 0.072 | 0.003 | 0.152 | 0.163 | 0 | 0.163 | 0 | 0.036 | 0.116 |
| **HAI** | **HEW22** | **July** | 0.034 | 0 | 0.188 | 0.456 | 0 | 0.456 | 0.017 | 0.037 | 0.134 |
| **HAI** | **HEW36** | **July** | 0.107 | 0 | 0.065 | 0.059 | 0.059 | 0 | 0.006 | 0.059 | 0 |
| **HAI** | **HEW47** | **July** | 0.164 | 0.009 | 0.017 | 0.230 | 0.011 | 0.218 | 0 | 0.017 | 0 |
| **SCH** | **SEW1** | **July** | 0.561 | 0 | 0 | 0.018 | 0 | 0.018 | 0 | 0 | 0 |
| **SCH** | **SEW2** | **July** | 0.255 | 0 | 0.021 | 0 | 0 | 0 | 0.021 | 0 | 0 |
| **SCH** | **SEW3** | **July** | 0.325 | 0 | 0.087 | 0 | 0 | 0 | 0.079 | 0.008 | 0 |
| **SCH** | **SEW4** | **July** | 0.176 | 0 | 0.007 | 0.029 | 0 | 0.029 | 0 | 0.007 | 0 |
| **SCH** | **SEW5** | **July** | 0.270 | 0.003 | 0.141 | 0.087 | 0 | 0.087 | 0 | 0.141 | 0 |
| **SCH** | **SEW6** | **July** | 0.139 | 0 | 0.031 | 0.097 | 0 | 0.097 | 0 | 0.031 | 0 |
| **SCH** | **SEW7** | **July** | 0.260 | 0 | 0.128 | 0.156 | 0 | 0.156 | 0 | 0.128 | 0 |
| **SCH** | **SEW8** | **July** | 0.111 | 0 | 0.026 | 0.009 | 0.009 | 0 | 0.023 | 0.003 | 0 |
| **SCH** | **SEW9** | **July** | 0.283 | 0 | 0.026 | 0.140 | 0 | 0.140 | 0 | 0.026 | 0 |
| **SCH** | **SEW18** | **July** | 0.639 | 0 | 0.006 | 0 | 0 | 0 | 0 | 0.006 | 0 |
| **SCH** | **SEW35** | **July** | 0.411 | 0 | 0.315 | 0.015 | 0 | 0.015 | 0 | 0.315 | 0 |
| **SCH** | **SEW36** | **July** | 0.357 | 0.003 | 0.035 | 0.788 | 0 | 0.788 | 0 | 0.035 | 0 |
| **SCH** | **SEW37** | **July** | 0.254 | 0.003 | 0.268 | 0.120 | 0 | 0.120 | 0.032 | 0.236 | 0 |
| **SCH** | **SEW41** | **July** | 0.295 | 0 | 0.090 | 0 | 0 | 0 | 0 | 0.090 | 0 |
| **SCH** | **SEW43** | **July** | 0.211 | 0.018 | 0.342 | 0 | 0 | 0 | 0.035 | 0.307 | 0 |
| **SCH** | **SEW48** | **July** | 0.139 | 0 | 0.013 | 0.104 | 0.104 | 0 | 0 | 0.013 | 0 |
| **ALB** | **AEW4** | **November** | na | na | na | na | na | na | na | na | na |
| **ALB** | **AEW5** | **November** | na | na | na | na | na | na | na | na | na |
| **ALB** | **AEW6** | **November** | na | na | na | na | na | na | na | na | na |
| **ALB** | **AEW7** | **November** | na | na | na | na | na | na | na | na | na |
| **ALB** | **AEW8** | **November** | na | na | na | na | na | na | na | na | na |
| **ALB** | **AEW9** | **November** | na | na | na | na | na | na | na | na | na |
| **ALB** | **AEW17** | **November** | na | na | na | na | na | na | na | na | na |
| **ALB** | **AEW18** | **November** | na | na | na | na | na | na | na | na | na |
| **ALB** | **AEW25** | **November** | na | na | na | na | na | na | na | na | na |
| **ALB** | **AEW26** | **November** | na | na | na | na | na | na | na | na | na |
| **ALB** | **AEW27** | **November** | na | na | na | na | na | na | na | na | na |
| **ALB** | **AEW41** | **November** | na | na | na | na | na | na | na | na | na |
| **ALB** | **AEW42** | **November** | na | na | na | na | na | na | na | na | na |
| **ALB** | **AEW49** | **November** | na | na | na | na | na | na | na | na | na |
| **ALB** | **AEW50** | **November** | na | na | na | na | na | na | na | na | na |
| **HAI** | **HEW4** | **November** | na | na | na | na | na | na | na | na | na |
| **HAI** | **HEW5** | **November** | na | na | na | na | na | na | na | na | na |
| **HAI** | **HEW6** | **November** | na | na | na | na | na | na | na | na | na |
| **HAI** | **HEW10** | **November** | na | na | na | na | na | na | na | na | na |
| **HAI** | **HEW11** | **November** | na | na | na | na | na | na | na | na | na |
| **HAI** | **HEW12** | **November** | na | na | na | na | na | na | na | na | na |
| **HAI** | **HEW16** | **November** | na | na | na | na | na | na | na | na | na |
| **HAI** | **HEW17** | **November** | na | na | na | na | na | na | na | na | na |
| **HAI** | **HEW18** | **November** | na | na | na | na | na | na | na | na | na |
| **HAI** | **HEW21** | **November** | na | na | na | na | na | na | na | na | na |
| **HAI** | **HEW22** | **November** | na | na | na | na | na | na | na | na | na |
| **HAI** | **HEW23** | **November** | na | na | na | na | na | na | na | na | na |
| **HAI** | **HEW36** | **November** | na | na | na | na | na | na | na | na | na |
| **HAI** | **HEW40** | **November** | na | na | na | na | na | na | na | na | na |
| **HAI** | **HEW47** | **November** | na | na | na | na | na | na | na | na | na |
